# Prognostic value of systemic inflammation response index in patients with glioma: a meta-analysis

**DOI:** 10.3389/fimmu.2025.1576845

**Published:** 2025-05-23

**Authors:** Yizhen Jiang, Lin Zhou

**Affiliations:** ^1^ Department of Medical Oncology, Huzhou Central Hospital, Affiliated Central Hospital of Huzhou University, Huzhou, Zhejiang, China; ^2^ Clinical Laboratory, Zhejiang Xinda Hospital, Huzhou, Zhejiang, China

**Keywords:** systemic inflammation response index, glioma, meta-analysis, biomarker, prognosis

## Abstract

**Background:**

The systemic inflammation response index (SIRI) has been investigated for its prognostic relevance in patients with glioma; however, findings remain inconsistent. Therefore, this meta-analysis aimed to clarify the prognostic value of SIRI in glioma.

**Methods:**

PubMed, Web of Science, Embase, Cochrane Library, and CNKI were systematically searched through December 28, 2024. Pooled hazard ratios (HRs) with 95% confidence intervals (CIs) were calculated to assess the association between SIRI and glioma prognosis.

**Results:**

A total of 10 studies including 1,942 participants were analyzed. Elevated SIRI was significantly associated with poorer overall survival (OS) (HR=1.67, 95% CI=1.46–1.91, p<0.001) and shorter progression-free survival (PFS) (HR=1.80, 95% CI=1.29–2.52, p=0.001). Subgroup analyses indicated that the prognostic value of SIRI for OS and PFS was consistent regardless of sample size, pathological subtype, cutoff value, or type of survival analysis (p<0.05). Sensitivity and publication bias analyses confirmed the robustness of the results.

**Conclusion:**

This meta-analysis demonstrates that high SIRI is a significant predictor of OS and PFS in patients with glioma. SIRI may serve as a promising prognostic biomarker in glioma-related clinical practice.

## Introduction

Gliomas, tumors of the central nervous system (CNS) originating in glial cells, represent the most prevalent type of neurological tumor, accounting for approximately 80% of primary brain cancers (1). According to updated GLOBOCAN estimates, there were 321,476 new glioma cases and 248,305 related deaths worldwide in 2022 (2). Gliomas are classified into four grades based on genetic profiles and histopathological characteristics: grades I–II are considered low-grade gliomas, while grades III–IV are classified as malignant or high-grade gliomas (3, 4). Despite advances in glioma diagnosis and treatment, clinical outcomes remain poor, with a median survival of 14.5 months (1). Glioblastoma (GBM), a grade IV glioma, is the most prevalent, aggressive, and malignant primary brain tumor in adults (5). GBM accounts for 57.3% of all glioma cases and represents the most common histological subtype. Its prognosis is especially poor, with a 5-year survival rate of less than 6.9% (6). The identification of prognostic biomarkers is critical for improving clinical outcomes in patients with glioma (7). Therefore, there is an urgent need to identify novel, reliable biomarkers to predict glioma prognosis.

Over the past few decades, numerous studies have emphasized the pivotal role of the immune system and cancer-related inflammation in tumor initiation, progression, and metastasis (8, 9). The systemic inflammation response index (SIRI) has emerged as a prognostic tool that incorporates routine hematological parameters (10). Initially proposed in 2016, SIRI is calculated using the following formula: SIRI = neutrophil × monocyte/lymphocyte (10). SIRI has demonstrated significant prognostic value in various cancers, including colorectal cancer (11), gastric cancer (12), cholangiocarcinoma (13), thyroid cancer (14), and non-small cell lung cancer (NSCLC) (15). Its prognostic value in patients with glioma has also been explored (16–25); however, findings have been inconsistent. Some studies have reported that elevated SIRI is significantly associated with poor prognosis in patients with glioma (16–18, 20, 21). Whereas others have found no significant association between SIRI and glioma survival outcomes (19, 24). Consequently, the present study aimed to clarify the prognostic value of SIRI in patients with glioma.

## Materials and methods

### Study guideline

This meta-analysis was conducted in accordance with the Preferred Reporting Items for Systematic Reviews and Meta-Analyses (PRISMA) guidelines (26).

### Literature search

We systematically searched PubMed, Web of Science, Embase, Cochrane Library, and CNKI databases through December 28, 2024. The search strategy included the following terms: (systemic inflammation response index OR system inflammation response index OR SIRI OR systemic inflammatory response index) AND (glioma OR gliomas OR glioblastoma). No language restrictions were applied. Additionally, we manually reviewed the references of all included articles to ensure comprehensive coverage.

### Inclusion and exclusion criteria

Studies were included if they met the following criteria (1): glioma was pathologically confirmed (2); the study evaluated the association between SIRI and glioma prognosis (3); hazard ratios (HRs) with 95% confidence intervals (CIs) were reported (4); a defined threshold for stratifying high vs. low SIRI was provided; and (5) there were no language restrictions. Exclusion criteria were as follows (1): reviews, case reports, conference abstracts, letters, or commentaries (2); studies lacking survival outcome data (3); studies with duplicate participant cohorts; and (4) animal studies.

### Data extraction and quality assessment

Two independent reviewers (Y.J. and L.Z.) extracted data from eligible studies. Discrepancies were resolved through discussion. Extracted data included: first author, publication year, country, sample size, sex distribution, age, study design, study center type, WHO tumor grade, study period, tumor pathology, treatment modality, SIRI threshold and its determination method, follow-up duration, survival endpoints, type of survival analysis, HRs, and 95% CIs. The primary and secondary outcomes were overall survival (OS) and progression-free survival (PFS), respectively. Study quality was assessed using the Newcastle-Ottawa Scale (NOS) (27), with scores ranging from 0 to 9. Studies scoring ≥6 were considered high quality.

### Statistical analysis

To assess the prognostic value of SIRI in patients with glioma, pooled HRs and corresponding 95% CIs were calculated. Between-study heterogeneity was evaluated using Cochran’s Q test and the I^2^ statistics. An I^2^ > 50% and p < 0.10 indicated significant heterogeneity, in which case a random-effects model was applied; otherwise, a fixed-effects model was used. Subgroup analyses were performed based on various study characteristics to explore the prognostic relevance of SIRI. Sensitivity analyses were conducted by sequentially removing each study and recalculating the overall effect size to evaluate the robustness of the results. Publication bias was assessed using funnel plots as well as Begg’s and Egger’s tests. All statistical analyses were performed using Stata version 12.0 (StataCorp, College Station, TX, USA). A p-value <0.05 is considered statistically significant.

## Results

### Literature search process

**Figure 1:**
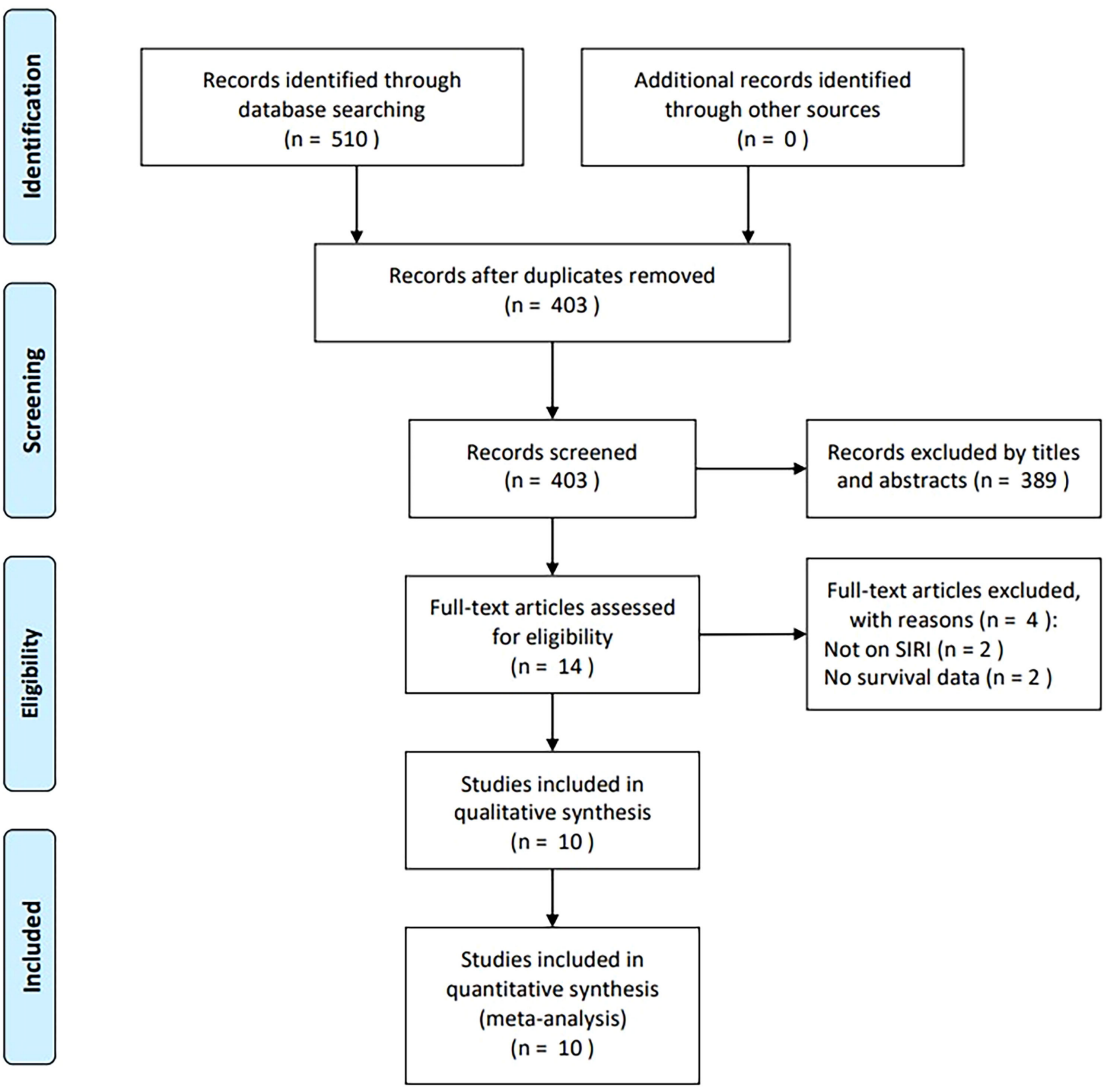
PRISMA flow diagram of study selection.

The initial database search identified 510 articles, of which 403 remained after the removal of duplicates (**Figure 1**). After screening titles and abstracts, 389 records were excluded due to irrelevance or being animal studies. Full texts of 14 articles were assessed for eligibility, and four were excluded—two for not evaluating SIRI and two for lacking survival data. Ultimately, 10 studies comprising 1,942 participants were included in the meta-analysis (16–25) (**Figure 1**).

### Characteristics of included studies

The 10 included studies were published between 2018 and 2024 and all employed retrospective designs (16–25) (**Table 1**). Six studies were conducted in China (16, 19, 21, 22, 24, 25), two in Turkey (17, 23), one in France (18) and one in Poland (20). Eight articles were published in English (17–24) and two in Chinese (16, 25). Sample sizes ranged from 80 to 358 participants (median: 189.5). Nine studies were single-center investigations (16–23, 25), while one was a multicenter study (24). Five studies enrolled participants with glioma (16, 19, 20, 24, 25), and five focused on participants with GBM (17, 18, 21–23). Treatment approaches included surgery in five studies (16, 20, 22, 24, 25) and multimodal therapy in five others (17–19, 21, 23). Reported SIRI thresholds ranged from 0.67 to 3.03 (median, 1.47). Nine studies determined cut-off values using receiver operating characteristic (ROC) curve analysis (16–21, 23–25), while one used X-tile software (22). HRs and 95% CIs were derived via univariate regression in five studies (18–21, 25) and multivariate regression in the remaining five (16, 17, 22–24). NOS scores ranged from 7 to 9, indicating high methodological quality (**Table 1**).

**Table 1:** Basic characteristics of included studies in this meta-analysis.

Study	Year	Country	Sample size	Gender (M/F)	Age (year) Median(range)	Study center	Study period	WHO grade	Pathology	Treatment	Cut-off value	Cut-off determination	Survival outcomes	Follow-up (month) Median(range)	Survival analysis	NOS score
Zhang, L. (16)	2018	China	80	49/31	36(12-82)	Single center	2006-2015	I-IV	Glioma	Surgery	0.67	ROC curve	OS, PFS	1-96	Multivariate	8
Topkan, E. (17)	2020	Turkey	181	116/65	59(24-80)	Single center	2007-2017	IV	GBM	Mixed	1.78	ROC curve	OS, PFS	1-120	Multivariate	8
Clavreul, A. (18)	2021	France	85	65/20	60(36-81)	Single center	2012-2020	IV	GBM	Mixed	2.55	ROC curve	OS, PFS	1-72	Univariate	8
He, Q. (19)	2021	China	105	57/48	50(18-79)	Single center	2013-2019	III-IV	Glioma	Mixed	1.26	ROC curve	OS	1-84	Univariate	7
Jarmuzek, P. (20)	2022	Poland	358	195/163	62.3(21.9-84.7)	Single center	2004-2021	I-IV	Glioma	Surgery	3.03	ROC curve	OS	7(1-123)	Univariate	8
Shi, X. (21)	2022	China	232	127/105	<65y: 193 ≥65y: 39	Single center	2014-2018	IV	GBM	Mixed	1.78	ROC curve	OS, PFS	1-80	Univariate	8
Wang, Z. (22)	2022	China	291	186/105	54(18-85)	Single center	2015-2019	IV	GBM	Surgery	1.26	X-tile	OS	1-54	Multivariate	7
Aydin, A. A. (23)	2024	Turkey	198	114/84	60(25-86)	Single center	2013-2022	IV	GBM	Mixed	1.62	ROC curve	OS, PFS	1-50	Multivariate	8
Liu, Z. Y. (24)	2024	China	246	153/93	54(23-80)	Multicenter	2017-2022	II-IV	Glioma	Surgery	1.10	ROC curve	OS	1-70	Multivariate	9
Zhao, S. (25)	2024	China	166	83/83	47	Single center	2015-2020	II-IV	Glioma	Surgery	1.32	ROC curve	OS	18-60	Univariate	8

M, male; F, female; WHO, World Health Organization; GBM, glioblastoma; OS, overall survival; PFS, progression-free survival; NOS, Newcastle-Ottawa Scale; ROC, receiver operating characteristic.

### SIRI and OS

**Figure 2:**
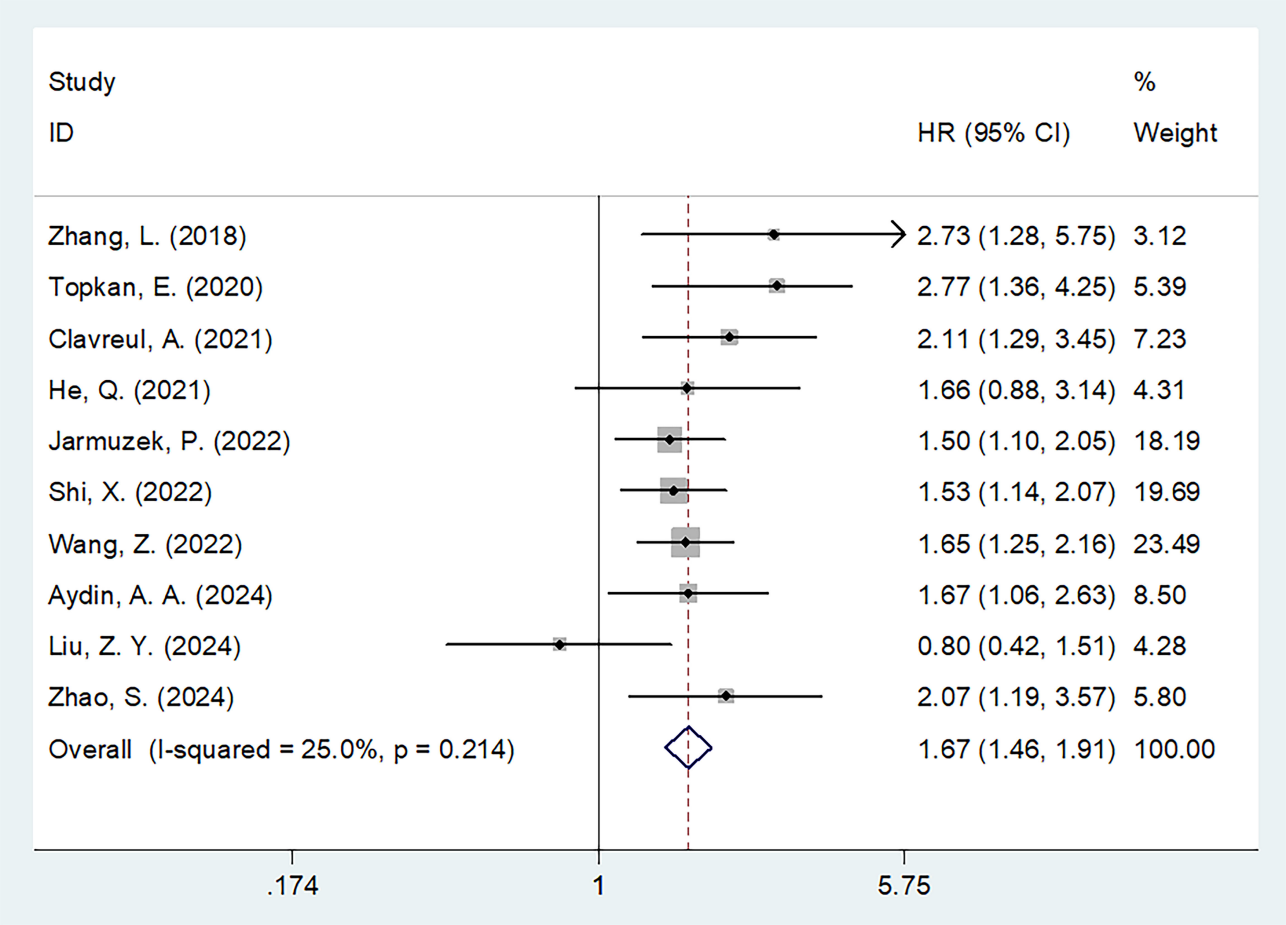
Forest plots of HR with 95% CI for correlation between SIRI and OS in patients with glioma.

All 10 studies (n=1,942 participants) examined the association between SIRI and OS in patients with glioma (16–25). Given the low heterogeneity among studies (I^2^ = 25.0%, p = 0.214), a fixed-effects model was applied. Pooled results revealed that elevated SIRI was significantly associated with worse OS (HR = 1.67, 95% CI = 1.46–1.91, p < 0.001) (**Figure 2**; **Table 2**). Subgroup analyses confirmed that SIRI was a significant prognostic factor for OS regardless of country, sample size, pathology, treatment modality, threshold, threshold determination method, or type of survival analysis (p < 0.05 for all; **Table 2**).

**Table 2:** Subgroup analysis of prognostic value of SIRI for OS in patients with glioma.

Subgroups	No. of studies	No. of patients	Effects model	HR (95%CI)	P	Heterogeneity	
						I^2^(%)	Ph
Total	10	1942	Fixed	1.67 (1.46-1.91)	<0.001	25.0	0.214
Country							
China	6	1120	Fixed	1.60 (1.35-1.90)	<0.001	32.7	0.191
Others	4	822	Fixed	1.78 (1.44-2.20)	<0.001	25.4	0.259
Sample size							
<190	5	617	Fixed	2.20 (1.70-2.86)	<0.001	0	0.779
≥190	5	1325	Fixed	1.52 (1.30-1.77)	<0.001	8.3	0.359
Study center							
Single center	9	1696	Fixed	1.73 (1.51-1.98)	<0.001	0	0.572
Multicenter	1	246	–	0.80 (0.42-1.52)	0.494	–	–
Pathology							
Glioma	5	955	Fixed	1.56 (1.25-1.95)	<0.001	46.2	0.115
GBM	5	987	Fixed	1.73 (1.47-2.04)	<0.001	0.6	0.403
Treatment							
Surgery	5	1141	Fixed	1.59 (1.33-1.90)	<0.001	46.7	0.112
Mixed	5	801	Fixed	1.77 (1.46-2.16)	<0.001	0	0.426
Cut-off value							
<1.50	5	888	Fixed	1.64 (1.33-2.02)	<0.001	45.2	0.121
≥1.50	5	1054	Fixed	1.69 (1.42-2.01)	<0.001	13.9	0.325
Cut-off determination							
ROC curve	9	1651	Fixed	1.68 (1.44-1.95)	<0.001	33.2	0.152
X-tile	1	291	–	1.65 (1.25-2.16)	<0.001	–	–
Survival analysis types							
Univariate	5	946	Fixed	1.65 (1.38-1.97)	<0.001	0	0.698
Multivariate	5	996	Fixed	1.70 (1.39-2.07)	<0.001	58.9	0.045

SIRI, systemic inflammation response index; GBM, glioblastoma; OS, overall survival; ROC, receiver operating characteristic.

### SIRI and PFS

**Figure 3:**
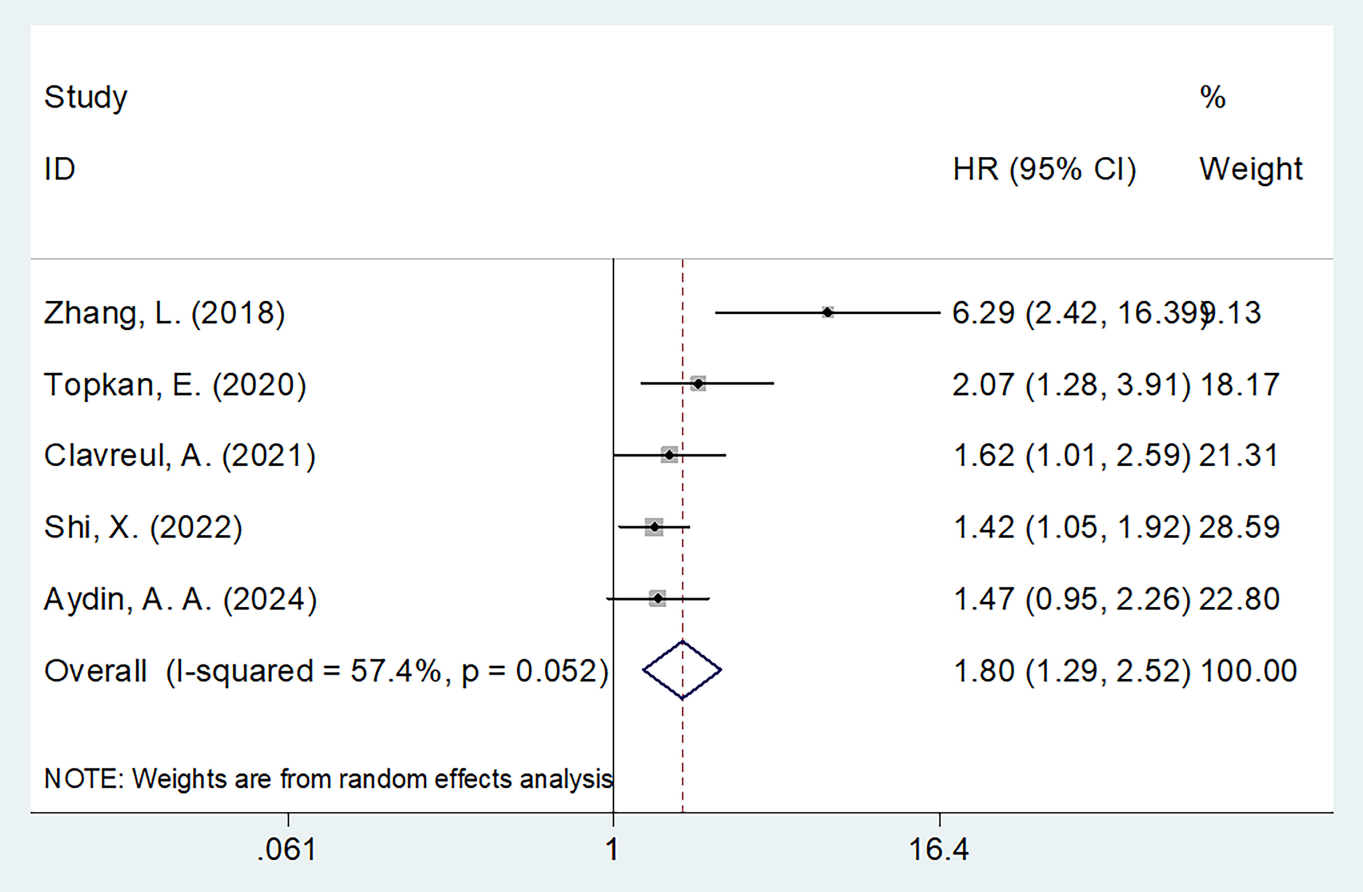
Forest plots of HR with 95% CI for correlation between SIRI and PFS in patients with glioma.

Five studies involving 776 participants assessed the relationship between SIRI and PFS (16–18, 21, 23). A random-effects model was used due to substantial heterogeneity (I^2^ = 57.4%, p = 0.052) (**Table 3**), likely arising from variations in sample size, pathology type, and treatment protocols across studies (**Table 1**). The meta-analysis showed that high SIRI was significantly associated with poorer PFS (HR = 1.80, 95% CI = 1.29–2.52, p = 0.001) (**Figure 3**; **Table 3**). Subgroup analyses demonstrated that this association remained significant regardless of sample size, pathology, threshold, or analysis method (p < 0.05 for all; **Table 3**).

**Table 3:** Subgroup analysis of prognostic value of SIRI for PFS in patients with glioma.

Subgroups	No. of studies	No. of patients	Effects model	HR (95%CI)	P	Heterogeneity	
						I^2^(%)	Ph
Total	5	776	Random	1.80 (1.29-2.52)	0.001	57.4	0.052
Country							
China	2	312	Random	2.78 (0.65-11.87)	0.167	88.2	0.004
Others	3	464	Fixed	1.65 (1.25-2.18)	<0.001	0	0.628
Sample size							
<190	3	346	Random	2.44 (1.29-4.64)	0.006	67.8	0.045
≥190	2	430	Fixed	1.43 (1.12-1.83)	0.004	0	0.907
Pathology							
Glioma	1	80	–	6.29 (2.41-16.39)	<0.001	–	–
GBM	4	696	Fixed	1.54 (1.26-1.89)	<0.001	0	0.692
Cut-off value							
<1.50	1	80	–	6.29 (2.41-16.39)	<0.001	–	–
≥1.50	4	696	Fixed	1.54 (1.26-1.89)	<0.001	0	0.692
Survival analysis types							
Univariate	2	317	Fixed	1.47 (1.15-1.90)	0.003	0	0.643
Multivariate	3	459	Random	2.37 (1.20-4.70)	0.014	73.2	0.024

SIRI, systemic inflammation response index; GBM, glioblastoma; PFS, progression-free survival; ROC, receiver operating characteristic.

### Sensitivity analysis

**Figure 4:**
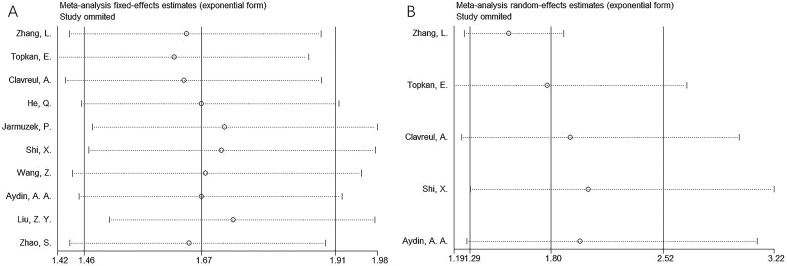
Sensitivity analysis. **(A)** OS and **(B)** PFS.

Sensitivity analyses were conducted by sequentially excluding each study to evaluate the stability of the pooled estimates. Results indicated that no single study significantly influenced the overall findings, confirming the robustness of the results for both OS and PFS (**Figure 4**).

### Publication bias

**Figure 5:**
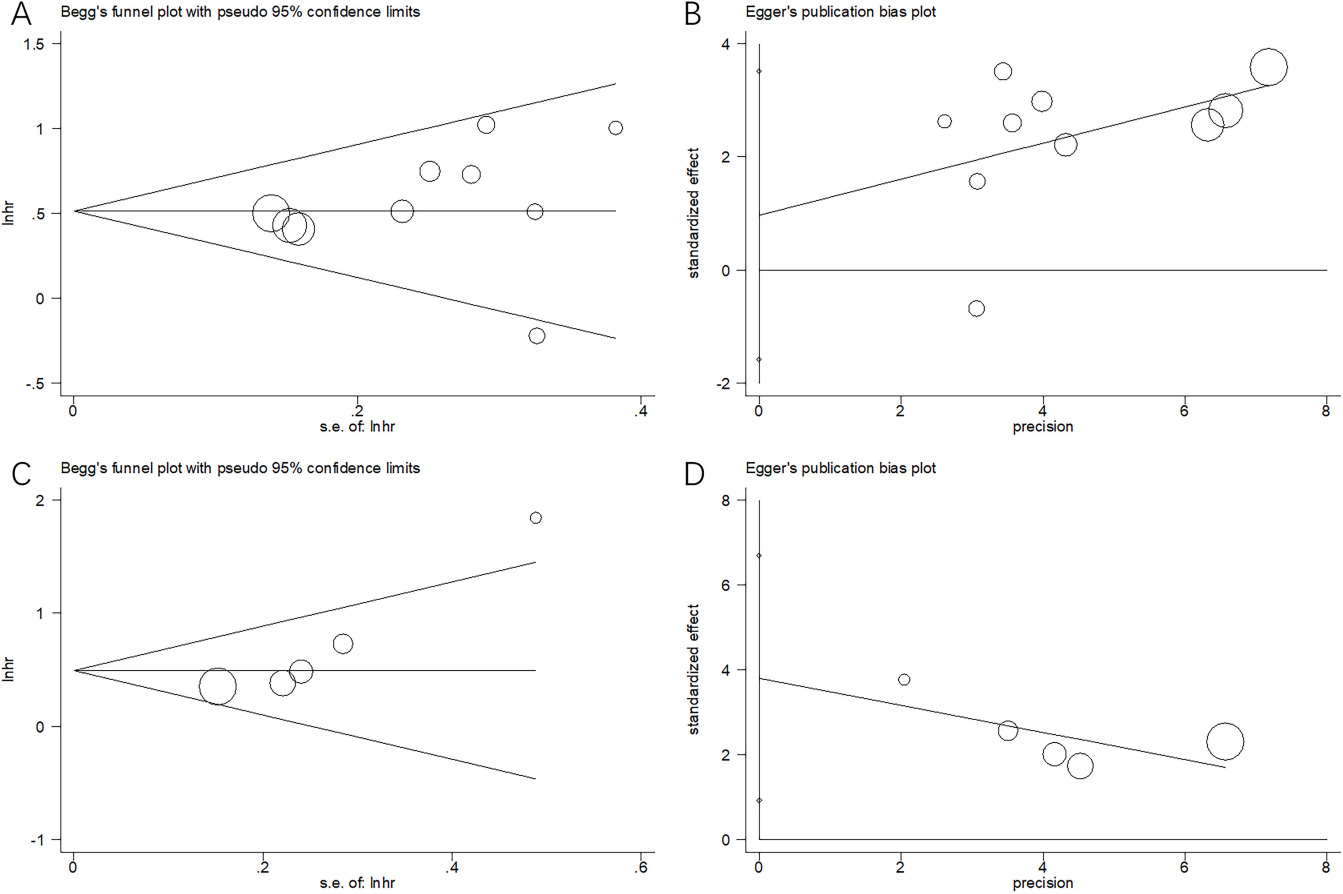
Publication bias by Begg’s test and Egger’s test. **(A)** Begg’s test for OS, p=0.371; **(B)** Egger’s test for OS, p=0.408; **(C)** Begg’s test for PFS, p=0.127; and **(D)** Egger’s test for PFS, p=0.225.

Potential publication bias was assessed using funnel plots, as well as Begg’s and Egger’s tests. The funnel plots were symmetrical (**Figure 5**), and statistical tests did not indicate significant publication bias for OS or PFS (Begg’s test: p = 0.371/0.408; Egger’s test: p = 0.127/0.225) (**Figure 5**).

## Discussion

SIRI has been extensively investigated for its prognostic value in glioma; however, previous findings remain inconsistent. The present study synthesized data from 10 studies involving 1,942 participants (16–25) to clarify the prognostic role of SIRI in glioma. Our results demonstrate that elevated SIRI significantly predicts both OS and PFS in individuals with glioma. Moreover, the prognostic performance of SIRI was consistent regardless of sample size, pathological classification, threshold definition, or type of survival analysis employed. Sensitivity analysis and assessments for publication bias confirmed the robustness of our findings. Collectively, these results identify SIRI as a meaningful prognostic marker for both short- and long-term outcomes in patients with glioma. To our knowledge, this study provides the first comprehensive evidence supporting the clinical relevance of SIRI in glioma prognosis.

SIRI is calculated using neutrophil, monocyte, and lymphocyte counts (10). An elevated SIRI may result from increased neutrophils and/or monocytes and/or decreased lymphocytes. Although the mechanisms underlying SIRI’s prognostic value in glioma remain to be fully elucidated, several plausible biological explanations exist. Neutrophils, as primary mediators of the inflammatory response, release vascular endothelial growth factor (VEGF), proteases, and chemokines that promote angiogenesis, thereby creating a tumor-supportive microenvironment (28). VEGF and interleukin-8 (IL-8), produced by tumor cells, further stimulate neutrophils within the tumor microenvironment, leading to the release of fibroblast growth factor, platelet-derived growth factor, matrix metalloproteinases, and interleukin-6 (IL-6) (29). In addition, neutrophils can suppress antitumor immunity by inhibiting T-cell activation through the production of reactive oxygen species, nitric oxide, and arginase (30). Monocytes, particularly those differentiating into tumor-associated macrophages (TAMs), also contribute to immune suppression and tumor progression. TAMs promote apoptosis of antitumor T cells and support angiogenesis through the release of pro-angiogenic factors (31). They also facilitate extracellular matrix degradation and tumor cell migration, thereby promoting metastasis (32). TAMs are recruited and activated by cytokines and chemokines such as tumor necrosis factor-α and monocyte chemoattractant protein-1 within the tumor microenvironment (33). The interaction between TAMs and cancer cells further enhances tumor angiogenesis, invasion, and migration while suppressing anticancer immune responses, ultimately contributing to disease progression and poor prognosis (34). Conversely, lymphocytes play a central role in host antitumor immunity. They contribute to cytotoxic responses that inhibit tumor growth and metastasis (35). Lymphocytes exert antitumor effects by activating the p53 signaling pathway and secreting IL-17, which induces cancer cell death and suppresses tumor proliferation (36). Furthermore, lymphocytes aid in immune surveillance by promoting cytotoxic cell-mediated destruction of tumor cells and preventing tumor dissemination (37). Therefore, SIRI represents a biologically plausible and clinically relevant prognostic marker derived from neutrophil, monocyte, and lymphocyte counts.

Numerous recent studies have reported the prognostic significance of SIRI in various cancers through meta-analyses (38–42). For instance, Shen et al. found that elevated SIRI significantly predicted both OS and PFS in patients with pancreatic cancer, based on a meta-analysis involving 1,160 participants (38). Similarly, Wu et al. reported that higher SIRI was strongly associated with poorer OS and disease-free survival in patients with gastric cancer, as shown in their meta-analysis of seven studies (39). A recent meta-analysis involving 3,728 participants demonstrated that elevated SIRI was significantly associated with both OS and PFS in NSCLC (40). Gu et al. also reported that high SIRI was markedly correlated with worse OS and PFS in patients with cancer treated with programmed cell death 1/PD-1 ligand 1 immune checkpoint inhibitors, based on their meta-analysis of six studies (41). In another meta-analysis involving 17 studies, Yang and colleagues showed that elevated SIRI was a significant predictor of poor OS in patients with oral cancer (42). These findings are consistent with our meta-analysis, further supporting the prognostic value of SIRI across a range of cancer types.

Despite the promising results, several limitations should be acknowledged. First, only retrospective studies were included, which may introduce heterogeneity. Second, the cut-off values used to define high SIRI were not standardized across studies, potentially leading to selection bias. Third, most included studies were conducted at single centers. Therefore, large-scale, prospective, multicenter studies are warranted to validate our findings.

## Conclusions

In summary, this meta-analysis demonstrated that elevated SIRI significantly predicted both OS and PFS in participants with glioma. Moreover, subgroup analyses confirmed the consistency of this prognostic effect across various study characteristics. These findings suggest that SIRI may serve as a promising glioma-related clinical prognostic biomarker.

## Data Availability

The original contributions presented in the study are included in the article/supplementary material. Further inquiries can be directed to the corresponding author.
